# Bench-to-Bedside Perspectives on the Development of Soluble TCR-Based Protein Biologics: Current Landscape, Control Strategy and Future Direction

**DOI:** 10.3390/ijms27198859

**Published:** 2026-10-04

**Authors:** Jingyi Zhang, Hongxin An, Chuanfei Yu, Weiyu Li, Meng Li, Lan Wang

**Affiliations:** 1 School of Life Science and Bio-pharmaceutics, Shenyang Pharmaceutical University, Shenyang 110016, China; 2 State Key Laboratory of Drug Regulatory Science, NHC Key Laboratory of Research on Quality and Standardization of Biotech Products, NMPA Key Laboratory for Quality Research and Evaluation of Biological Products, National Institutes for Drug Control, Beijing 102629, China

**Keywords:** soluble TCR-based protein biologic, T cell receptor -T cell engagers (TCR-TCE), bispecific antibody, clinical trial, nonclinical trial, quality control

## Abstract

Soluble T-cell receptor (TCR)-based biologics (protein-based) are an emerging class of immunotherapeutic agents that recognize antigenic peptides presented by the human leukocyte antigen (HLA) on the surface of target cells. Furthermore, they simultaneously bind to cluster of differentiation 3 (CD3) subunits on T cells, recruit T cells to target cells, and promote T-cell activation and degranulation, ultimately eliminating tumor cells. Soluble TCR-based protein biologics target intracellular antigens that are inaccessible to conventional antibody-based drugs. Consequently, the development of soluble TCR-based protein biologics has generated significant interest. One soluble TCR-based protein biologic is already approved for marketing, and several other drugs are entering clinical stages. Soluble TCR-based protein biologics have substantial therapeutic benefits but may cause cytokine release syndrome (CRS), off-target toxicity, and drug resistance. This review outlines the structural characteristics and mechanisms of action of soluble TCR-based protein biologics, introduces nonclinical research concerning soluble TCR-based protein biologics, describes the clinical progress, discusses the quality control of TCR drugs, and explores the challenges and possible future improvements for soluble TCR-based protein biologics.

## 1. Introduction

Selective targeting of tumors can improve cancer treatment outcomes. The development of bispecific antibodies, T-cell redirecting technologies, and immunotherapies such as chimeric antigen receptor T-cell (CAR-T) therapy has revolutionized the treatment of malignant tumors [1,2,3]. Building on these immunotherapies and the engineering of T-cell receptor (TCR) structures, soluble TCR-based protein biologics have emerged as one of the most groundbreaking innovative drugs in recent years. Soluble TCR-based protein biologics are based on the TCR structure or TCR-like functional structures capable of recognizing antigenic peptide–human leukocyte antigen (pHLA), including TCR-T cell engagers (TCR-TCEs) and TCR mimic antibodies (TCRms). Soluble TCR-based protein biologics overcome the limitations of conventional immunotherapies, which are restricted to targeting surface antigens, by employing the mechanism for HLA presentation of intracellular antigenic peptides on the target cell surface. Thus, soluble TCR-based protein biologics expand the scope of target recognition. Soluble TCR-based protein biologics combine the ability to target tumor antigens with the immune effector functions of T cells to kill tumor cells. Thus, soluble TCR-based protein biologics can efficiently and continuously kill tumor cells [4,5].

**Figure 1:**
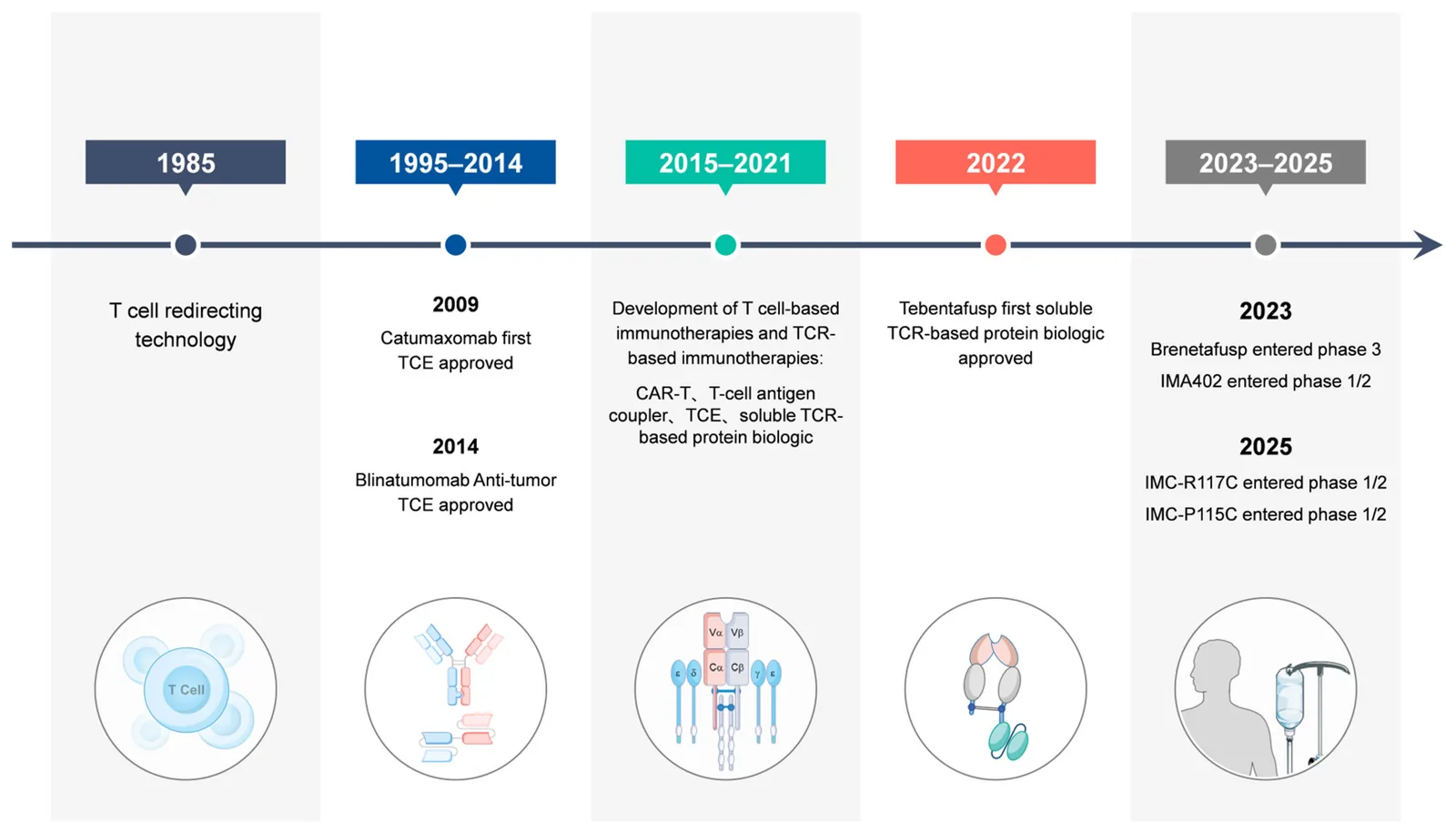
Developmental timeline and key milestones for soluble TCR-based protein biologics. Over the past four decades, advances in T cell-redirecting technologies have led to the emergence of various T cell-based therapeutics. Among them, soluble TCR-based protein biologics have progressed rapidly over the past five years, with several agents advancing into different phases of clinical development. TCR, T cell receptor; TCE, T cell engagers; CAR-T, chimeric antigen receptor T-cell.

The developmental timeline and key milestones of soluble TCR-based protein biologics are shown in Figure 1. T cell engagers (TCEs) were developed before soluble TCR-based protein biologics. Conventional TCEs simultaneously bind to tumor-associated antigens (TAAs) directly expressed on the tumor cell surface and to CD3 subunits on T cells, thereby redirecting T cells to tumor sites and inducing tumor cell killing [6]. Catumaxomab, which was approved in 2009, is the first TCE drug used to treat malignant ascites caused by advanced cancer [7]. Blinatumomab, which was approved in 2014, is a groundbreaking TCE targeting CD19 on B cells. Blinatumomab is used to treat acute lymphoblastic leukemia [8,9]. The development of blinatumomab marked an important milestone in TCE development. Research on TCEs against various tumor-associated targets in hematologic malignancies and solid tumors has grown [10]. The selection of targets has shifted from B-cell antigens to TAAs. A wave of TCE approvals occurred between 2022 and 2025, and over 200 TCEs are now in clinical studies [6,11].

Building upon TCEs, engineering of soluble, high-affinity TCRs led to the development of TCR-TCEs, a class of soluble TCR-based protein biologics with distinct advantages. The first soluble TCR-based protein biologic to be approved for marketing was tebentafusp, which was developed by Immunocore. Tebentafusp (trade name Kimmtrak) was approved by the FDA for treating uveal melanoma in January 2022 and subsequently approved by the European Medicines Agency in April 2022. Tebentafusp is marketed in the UK, Brazil, Canada, and Australia. Soluble TCR-based protein biologics are under development for an increasing number of indications. As of December 2025, 76 soluble TCR-based protein biologics are at various stages of development, ranging from preclinical to Phase III clinical trials, and 11 drugs have entered the clinical stage (Table 1). Most of the newly developed drugs target cancers. For example, brenetafusp, which was developed by Immunocore, is in Phase III clinical trials for the treatment of melanoma, and IMC-M113V, which was also developed by Immunocore, is in Phase I/II clinical trials for the treatment of HIV infection. Another type of soluble TCR-based protein biologic, TCRm, mimics the mechanism of TCRs based on bispecific antibodies. Their intrinsic effector functions offer multiple advantages and flexibility, including ease of protein engineering [12]. The development of TCRms is currently in the preclinical and early clinical research stages. Overall, the entry of soluble TCR-based protein biologics into the global market is accelerating, and these drugs hold broad research prospects.

The soluble TCR-based protein biologic field has gained significant momentum but is in its early stages. Key challenges still need to be overcome, including toxicity, immunosuppression, and drug resistance. Herein, we comprehensively outline the mechanism of action, structural characteristics, specific research status, and challenges faced by soluble TCR-based protein biologics. In addition, we propose quality control strategies for soluble TCR-based protein biologics and explore the future development of soluble TCR-based protein biologics.

## 2. Mechanisms of Action of Soluble TCR-Based Protein Biologics

### 2.1. Mechanisms of Action of TCR and T-Cell Immune Effects

TCRs are naturally occurring antigen sensors that detect, amplify, and coordinate cellular immune responses to cell surface and intracellular proteins after presentation to cells. TCR target proteins are selectively expressed by tumor cells, including viral antigens, neoantigens, overexpressed antigens, and differentiation antigens [13]. providing the basis for emerging immunotherapy for cancer. Unlike antibodies, which physiologically bind to the cell surface and soluble epitopes, TCRs recognize HLA-presented peptides derived from intracellular proteins without requiring the complete protein and can recognize a broader range of tumor targets [14].

**Figure 2:**
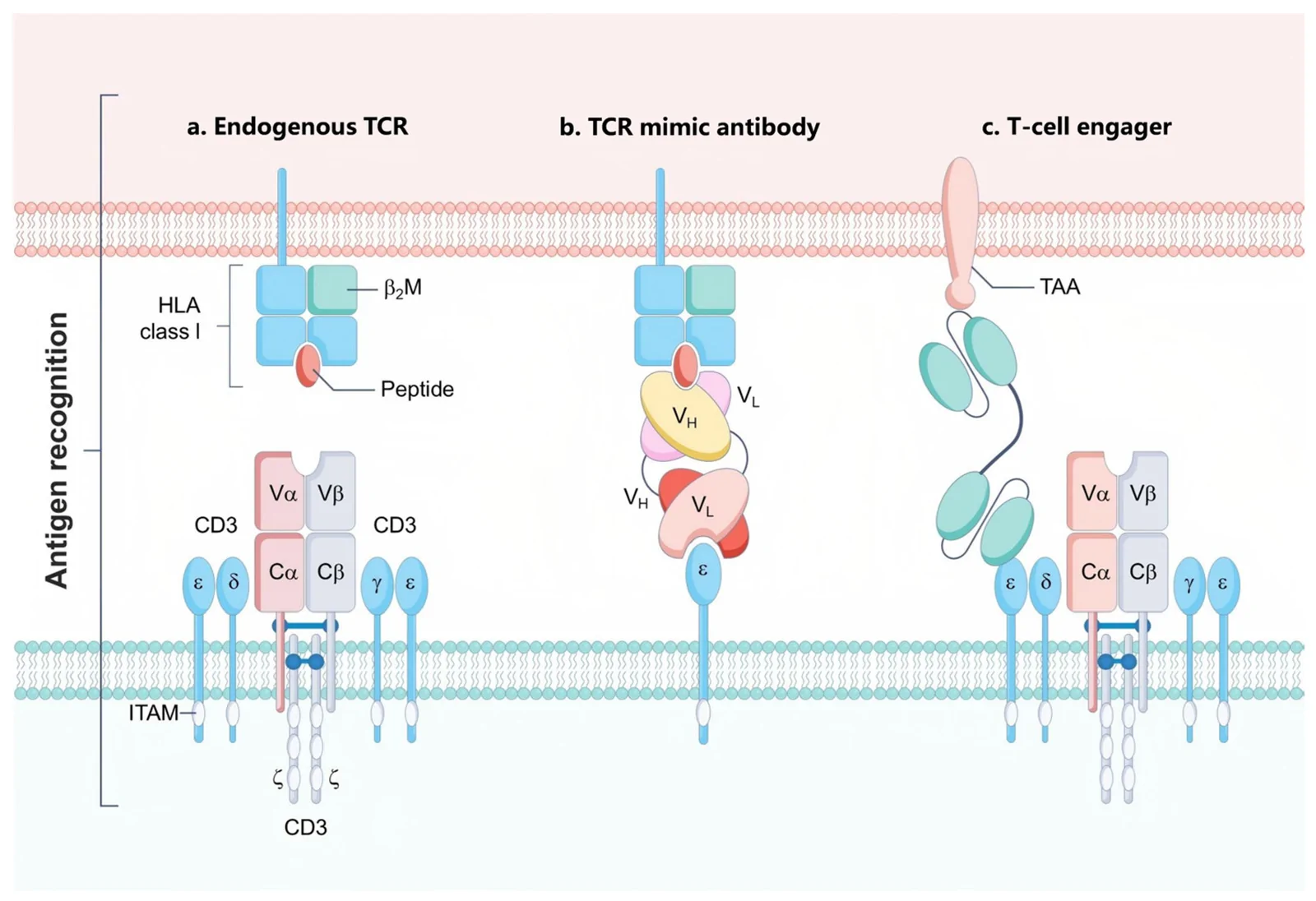
Molecular architecture of soluble T-cell receptor (TCR)-based protein biologics. Endogenous TCRα/TCRβ hemichains are covalently linked via a single interchain disulfide bond (**a**). Non-covalent interactions with the CD3 molecules facilitate intracellular signaling. TCR mimics antibodies (TCRms) are recombinant bispecific proteins that employ antibody VH/VL domains in place of a TCRα/TCRβ to engage a specific pHLA complex (**b**), an antibody-derived antigen-binding variable heavy chain, and a variable light chain specific for CD3ε. T-cell engagers (TCEs) are bispecific antibodies targeting tumor-associated antigen (TAA) and CD3ε (**c**). ITAM, immunoreceptor tyrosine-based activation motif; CD3, cluster of differentiation 3; pHLA, peptide–human leukocyte antigen.

The TCR-CD3 complex is a transmembrane octameric complex with functionally distinct subunits responsible for antigen recognition and signal transduction. The complex includes the TCRα and TCRβ chains, the constant CD3γε and CD3εδ heterodimers, and the CD3ζ ζ homodimer. The subunits assemble in a 1:1:1:1 stoichiometric ratio into the TCRαβ:CD3δε:CD3γε:CD3ζζ complex (Figure 2a). The CD3 subunits contain cytoplasmic immunoreceptor tyrosine-based activation motifs (ITAMs). Tyrosine phosphorylation of ITAMs is a critical step in T-cell activation [15,16,17]. Each TCR hemi-chain consists of an antigen-binding variable (V) region, a constant (C) region, a transmembrane domain, and a short non-signaling cytoplasmic tail. Based on the structure and signal transduction mechanisms of endogenous TCRs, TCEs and Soluble TCR-based protein biologics have been designed. The comparison of endogenous TCR, soluble TCR-based protein biologics, and TCEs is shown in Table 2. These agents share a common mechanism of action: one arm binds CD3 to redirect T cells. However, they differ in their antigen-targeting strategies. Conventional TCEs directly target antigens on the surface of target cells and do not inherently possess a TCR-like structure or the ability to target pHLA in a TCR-like manner. In contrast, Soluble TCR-based protein biologics, such as TCR-TCEs and TCRms, can target pHLA and thereby exhibit TCR-like targeting function. The comparison of their distinct targeting modalities is shown in Figure 2 and Table 2. The domains in these structures recognize membrane surface antigens and link to endogenous TCRs, independent of TCR recognition.

Major histocompatibility complexes (MHCs) can present specific intracellular peptides from tumor cells on the tumor cell surface as antigenic peptide–MHC (pMHC) complexes, which are recognized by TCRs, triggering T-cell immune effects and tumor killing. Human MHCs, termed human leukocyte antigen (HLAs), are divided into two classes: Class I (HLA-A/B/C) and Class II (HLA-DP/DQ/DR). HLA Class I molecules present endogenous peptides on the cell surface, forming peptide–HLA (pHLA) Class I complexes. The pHLA Class I complexes are recognized by CD8^+^ T cells, triggering cytotoxic T lymphocyte-mediated cytolysis. HLA Class II molecules present exogenous peptides on antigen-presenting cells (APCs), forming pHLA Class II complexes, which activate CD4^+^ T cells to regulate humoral immunity and inflammation [18,19,20]. The recognition of antigenic pHLA complexes by TCRs is crucial for immune system function but also initiates and drives disease occurrence and progression. Complete activation of T cells requires at least two signals. The first signal is from the antigen-specific recognition signal transmitted after TCR recognizes pHLA [16,21], activating T cells to release granzymes and perforin to kill tumor cells. The second signal is from the binding of co-stimulatory molecules on the T-cell surface (e.g., CD28 subfamily, TNF/TNFR subfamily, inducible T-cell co-stimulator) to corresponding ligands on the APC surface. The involvement of co-stimulatory molecules enables sustained T-cell activation, proliferation, and differentiation, which are necessary for an effective immune response [22].

### 2.2. Soluble TCR-Based Protein Biologics

#### 2.2.1. TCR-TCE

TCR-TCEs, which are engineered based on TCR structures, are soluble bispecific proteins composed of a TCR and an anti-CD3 antibody built on a traditional TCE. TCEs connect tumor cell antigens and the CD3 region of TCRs and exert tumor-targeting effects by recognizing TAAs on the surface of tumor cells [23], independent of HLA action. Although this strategy can overcome tumor cell escape from immune surveillance by downregulating HLA class I molecule expression [24], the range of recognizable targets is limited.

**Figure 3:**
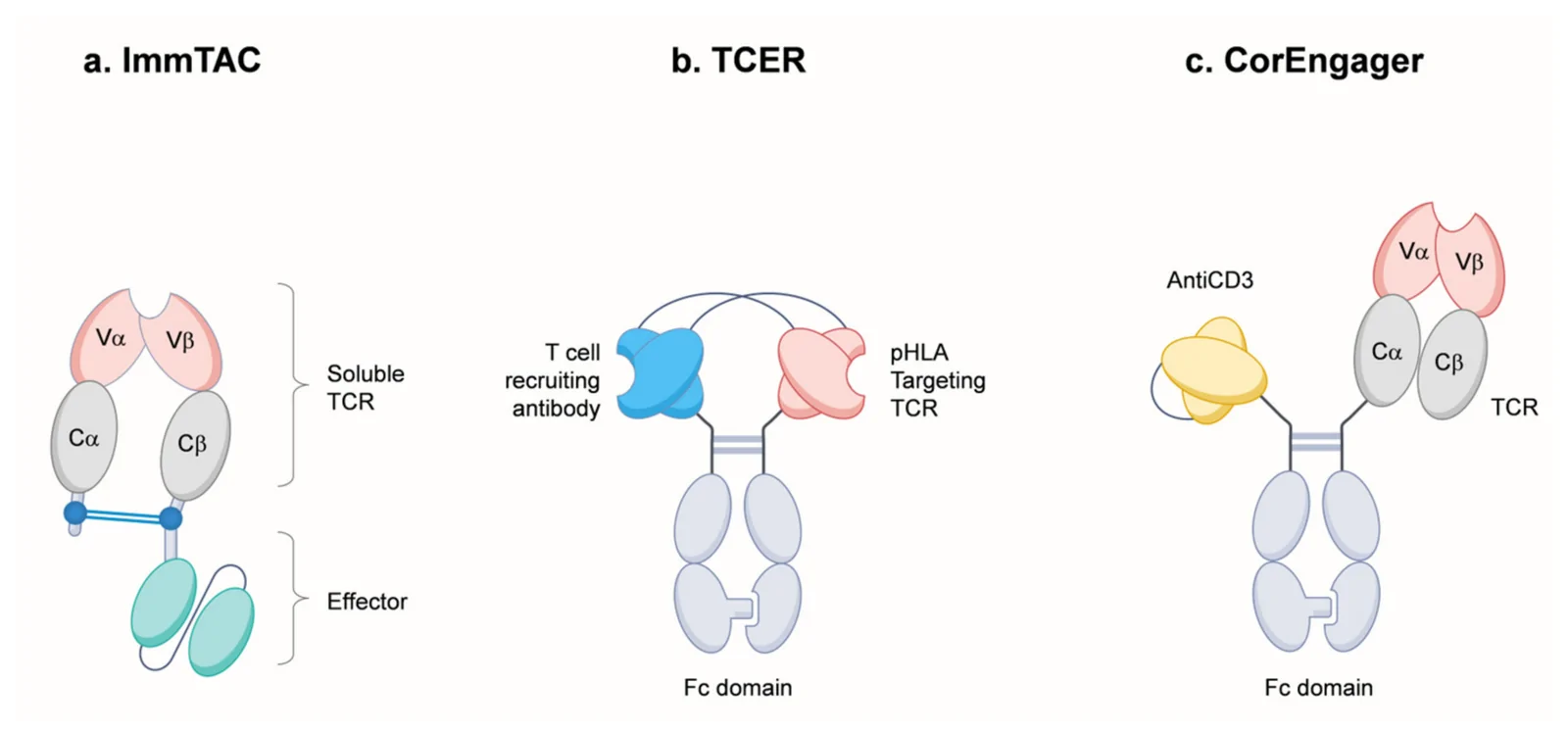
Structures of TCR -T cell engagers (TCR-TCEs) from different platforms. TCR-TCEs developed on the ImmTAC (**a**) platform consist of a high-affinity soluble TCR (red and light grey) linked by disulfide bonds (dark blue) and fused with an anti-CD3ε scFv (green). TCR-TCEs developed on the TCER (**b**) platform consist of the variable region of a TCR (red) connected to an anti-CD3 scFv (blue) and fused with an Fc domain (light grayish blue). TCR-TCEs developed on the CorEngager (**c**) platform consist of a full TCR structure connected to an anti-CD3 scFv (yellow) and fused with an Fc domain.

**Figure 4:**
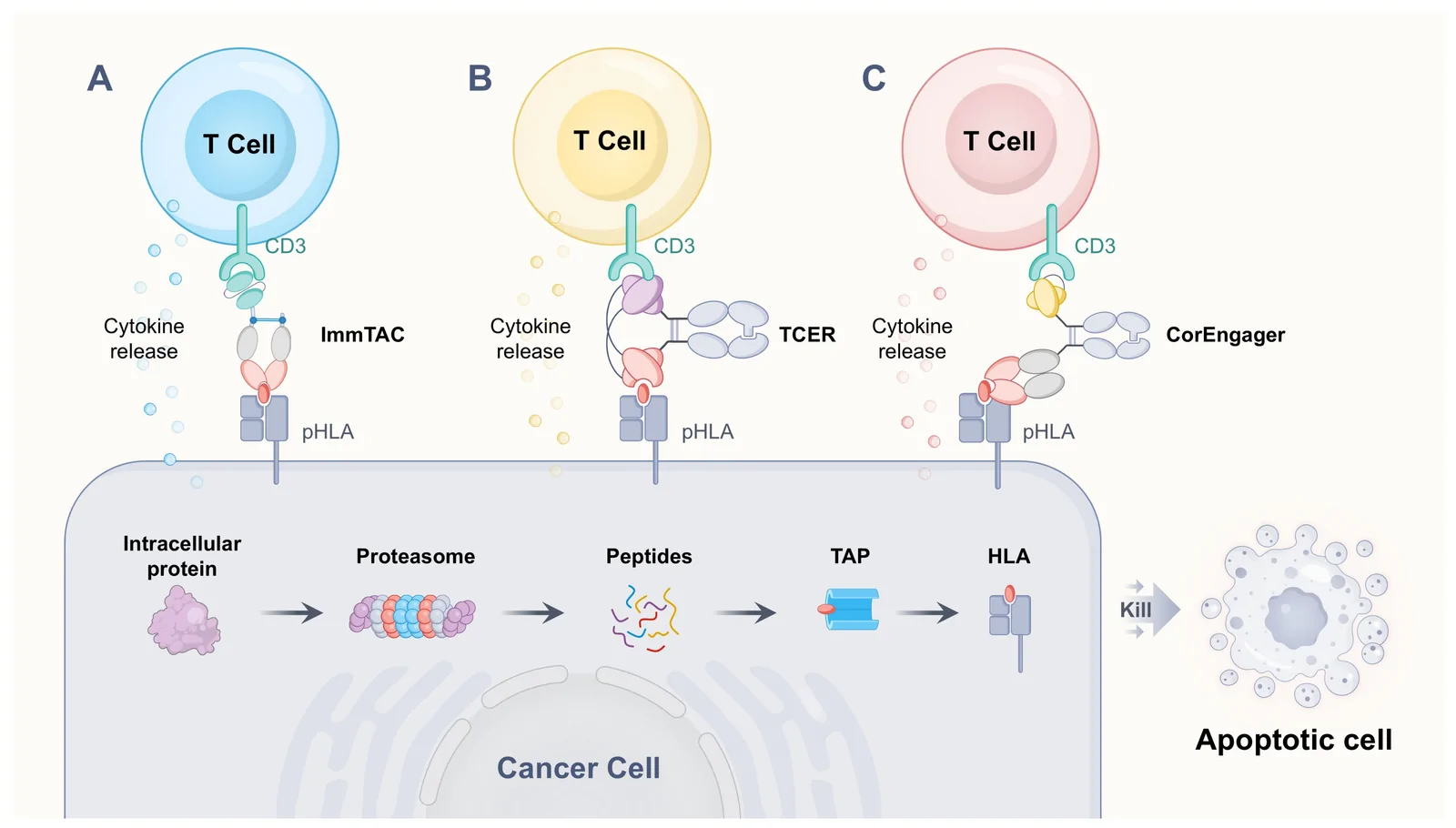
Mechanisms of action of soluble T-cell receptor (TCR)-based protein biologics. (**A**) ImmTAC. (**B**) TCER. (**C**) CorEngager. All three platforms share a similar mechanism of action. Intracellular proteins are processed and presented on the cell surface by human leukocyte antigen (HLA). Soluble TCR-based protein biologics recognize peptide–HLA (pHLA) via their high-affinity TCR targeting domain and recruit T cells via their anti-CD3 scFv domain. TAP, transporter associated with antigen processing.

Unlike traditional TCEs, TCR-TCEs are bifunctional proteins that use affinity-matured soluble TCRs or TCR-like antibodies as the tumor-targeting arm and an anti-CD3 scFv as the T-cell–activating arm. TCR-TCEs specifically recognize pHLA presented on the tumor cell surface and redirect polyclonal T cells to kill tumors. This mechanism of recognizing intracellular antigenic peptides presented by HLA expands the range of druggable targets for TCR-TCEs. Major platforms for developing TCR-TCEs include ImmTAC by Immunocore, TCER by Immatics, and CorEngager by CorreGene (Figure 3). ImmTAC removes the transmembrane domain of the natural TCR and the original interchain disulfide bond. An artificial disulfide bond is introduced by mutating two amino acid residues to cysteines near the membrane-proximal end [25] to generate a soluble, stable TCR. The TCR is then engineered to increase affinity, and the high-affinity soluble TCR is fused with an anti-CD3 scFv [26], creating a bridge between T cells and target cells. ImmTAC is the mainstream platform for the soluble TCR-based protein biologics in clinical research stages. Immunocore has also developed the ImmTAV platform for infectious diseases, aimed at developing treatments for chronic hepatitis B, HIV, and other persistent infections [27,28]. Using the TCER platform, a non-full-length TCR structure is selected and fused with the variable region of the TCR with an anti-CD3 scFv. A silenced Fc domain, which provides good stability and facilitates production, is added. TCR-TCEs engineered on the TCER platform have high affinity and exhibit activity even at low target expression levels [29]. In contrast to TCER, CorEngager incorporates a relatively complete TCR structure fused to an anti-CD3 scFv and an Fc domain. This engineered drug is designed to provide potentially favorable pharmacokinetic properties, tumor penetration ability, and CMC attributes, and may therefore offer a promising development profile. The mechanisms of action of soluble TCR-based protein biologics are shown in Figure 4.

#### 2.2.2. TCR Mimic Antibodies

TCRms are a class of engineered monoclonal antibodies that mimic the function of TCRs. TCRms recognize pHLA presented on the cell surface by HLA class I molecules. Thus, they target intracellular antigens inaccessible to conventional antibodies [30]. Although conventional antibodies are readily available, stable, inexpensive, and exhibit good druggability, they can only target cell membrane surface targets, such as CD19 or HER2 [31,32]. Small-molecule drugs can penetrate cell membranes and act inside cells, but may induce drug resistance and off-target toxicity [33]. TCRms provide an alternative way to indirectly recognize tumors. Similar to the mechanism for TCR recognition of intracellular tumor targets presented on pHLA complexes on the tumor cell surface, TCRms recognize antigens in the context of HLA, most commonly HLA-A*02:01, expanding the scope of target recognition [34,35]. TCRms are more sensitive to target cells than conventional antibodies and immunotherapies such as CAR-T. For example, TCRms can exert effective killing against cells presenting as few as 3–10 copies of *KRAS* G12V on the surface [36], approaching the physiological sensitivity of TCRs. Therefore, even if tumor cells severely downregulate antigen presentation and only a few pHLA complexes remain, TCRms may still be effective, offering new hope for overcoming immune escape.

### 2.3. Targets

Target selection is crucial to treating diseases. Tumor targets can be divided into TAAs and tumor-specific antigens (TSAs). TAAs include overexpressed antigens, such as WT1, PRAME, MAGE, and NY-ESO-1. [37,38,39,40]. TSAs include fusion proteins such as BCR-ABL [41], viral antigens such as HPV and HBV, and mutated antigens such as Ras, p53, and Myc [42]. TAAs are often chosen as targets for soluble TCR-based protein biologics. However, TAAs are not unique to tumor cells. TAAs are abnormally overexpressed or re-expressed in tumor cells but are also expressed in normal tissues. Examples include MART-1 in melanoma and carcinoembryonic antigen in many epithelial cancers, which are also expressed at low levels in normal melanocytes and fetal tissues, respectively [43,44]. Cancer-testis antigens, including NY-ESO-1, MAGE family, and PRAME, are usually silenced by methylation in adult tissues and are expressed only in the testis (an immune-privileged organ) and various tumors [45], making them ideal targets for soluble TCR-based protein biologic design. Moreover, the majority of TSAs that drive tumor initiation and progression reside within cells [42]. Therefore, designing drugs that recognize intracellular targets is very important. Neoantigens are also ideal targets. Neoantigens are peptides that are uniquely generated by tumor-specific mutations and are absent from the normal genome [46,47]. Targeting neoantigens reduces off-target toxicity and improves therapeutic outcomes. Neoantigens arise from non-synonymous single-nucleotide variations, frameshifts due to insertions/deletions, post-transcriptional modifications, and gene fusions [48,49]. Private neoantigens, consisting of each patient’s mutation profile and HLA type, are usually patient-specific [50]. Public neoantigens are peptides derived from driver gene mutations that occur frequently in a large number of patients (e.g., *KRAS* G12D) and are presented by a relatively common HLA subtype in the population (e.g., HLA-A*02:01) [51,52,53].

Selecting ideal targets, such as public neoantigens, is crucial for soluble TCR-based protein biologic design. In addition, accurately and rapidly screening patients most likely to benefit from a specific soluble TCR-based protein biologic therapy is also essential. For example, patient HLA typing and specific antigen expression are necessary for soluble TCR-based protein biologics targeting pHLA. HLA is highly polymorphic, with tens of thousands of alleles identified, and HLA allele frequencies differ markedly across populations [54]. A given soluble TCR-based protein biologic typically targets only one HLA subtype. For example, tebentafusp is restricted to HLA-A*02:01. This requires patients not only to express the target antigen (e.g., gp100) but also to carry the matching HLA subtype (e.g., HLA-A*02:01). Such subtype restriction can limit the clinical applicability of soluble TCR-based protein biologics. In addition, pHLA targets are subject to abundance constraints, and many are present at low copy numbers per cell. For instance, epitopes derived from *KRAS* G12V neoantigens may be present at <10 copies per cell [55]. Therefore, source antigen expression may not necessarily reflect the presentation of the corresponding pHLA complex on target cells.

Immunopeptidomics identifies and quantifies HLA-presented peptides through mass spectrometry (MS)-based methods [56,57]. For target discovery, immunopeptidomics can directly identify pHLA targets recognized by soluble TCR-based protein biologics and can uncover non-canonical antigens that may serve as potential targets for soluble TCR-based protein biologics. For specificity assessment, immunopeptidomics can systematically detect the cross-reactivity and off-target profile of soluble TCR-based protein biologics. Combined with machine learning, it can also predict and detect off-target pHLAs that share little or no sequence homology with the intended target yet may still bind to soluble TCR-based protein biologics [58]. For quantitative assessment, immunopeptidomics can quantify pHLA presentation levels, supporting the validation of in vivo efficacy and safety of soluble TCR-based protein biologics at physiological expression levels. In clinical translation, immunopeptidomics has been integrated into real-time antigen discovery pipelines and clinical trials for soluble TCR-based protein biologics [59]. Therefore, immunopeptidomics currently serves as a core experimental tool for target discovery and validation of soluble TCR-based protein biologics, compensating for the limitations of sequencing and computational predictions in confirming peptides that are both presented and immunogenic [60].

## 3. Nonclinical Research on Soluble TCR-Based Protein Biologics

Tebentafusp, approved by the FDA in 2022, is the only approved soluble TCR-based protein biologic. Thus, the development of this drug provides a basis for nonclinical research into soluble TCR-based protein biologics [61]. The nonclinical development of soluble TCR-based protein biologics should follow the FDA’s “Guidance for Industry: Bispecific Antibody Development Programs” (2021) [62] and the International Council for Harmonization (ICH) S9/S6(R1) [63,64], while fully considering the unique challenges posed by the pHLA targeting mechanism. Importantly, no TCR-specific regulatory guidance currently defines the full nonclinical package for soluble TCR-based protein biologics. Therefore, the recommendations below are classified as regulatory guidance, established practice from published TCR-TCE experience, or our proposed development strategy (Table 3).

### 3.1. Unique Attributes of Nonclinical Research on Soluble TCR-Based Protein Biologics

Soluble TCR-based protein biologics possess inherently unique attributes that distinguish them from conventional antibody drugs. These attributes should be considered during all aspects of nonclinical study design. The in vivo activity of soluble TCR-based protein biologics strictly depends on the correct processing and presentation of the tumor target antigen peptide and the co-expression of specific HLA alleles (e.g., HLA-A*02:01), and these processes may be species specific. Moreover, engineered soluble TCRs have very high binding affinity for pHLA [65], suggesting that even extremely low pHLA expression levels in normal tissues may pose a safety risk. Soluble TCR-based protein biologics utilize a high-affinity TCR end and a low-affinity anti-CD3 end, making T-cell activation highly dependent on pHLA targeting and reducing off-target risks [66]. For example, in preclinical mouse models, IMA401, a MAGE-A4/8- and CD3-targeting bispecific soluble TCR-based protein biologic, induced tumor regression and remission, and the low-affinity CD3-binding domain demonstrated better tumor control [29]. Soluble TCR-based protein biologics also exhibit diverse molecular formats and pharmacokinetic profiles, including soluble TCR-anti-CD3 fusion proteins without an Fc domain (short t_1_/_2_, approximately hours, requiring weekly dosing) and next-generation formats with extended half-lives via Fc fusion or albumin-binding domains (e.g., Immatics TCER^®^ platform [29], extended t_1_/_2_). The molecular format directly impacts the design of dosing regimens for toxicology studies.

### 3.2. Species Relevance Assessment

ICH S6(R1) provides general guidance for biotechnology-derived products, including antibody-like molecules, and recommends assessment of relevant species [63]. If a relevant species exists, safety evaluation should be conducted in that species; in the absence of a relevant species, alternative strategies should be adopted [63]. The species relevance of soluble TCR-based protein biologics depends on the pHLA recognition domain and the CD3-binding domain of each individual molecule. The mechanisms of peptidome processing and presentation differ among species, including mice and cynomolgus monkeys (approximately 40% of the mouse MHC-I peptidome overlaps with that of humans [67]). In addition, the species cross-reactivity of anti-CD3 binding domains is clone-dependent; some anti-CD3 binding domains have been shown to cross-react with human and cynomolgus monkey CD3ε [68], whereas mouse CD3ε is generally not recognized because of sequence divergence. Consequently, conventional animal species are often pharmacologically irrelevant. However, the species relevance of Soluble TCR-based protein biologics must still be assessed individually for each therapeutic molecule. If both functional arms are confirmed to lack a relevant animal species, appropriate alternative strategies should be considered. One option is to replace animal models with a comprehensive in vitro system; according to the in vitro nonclinical evaluation protocol for ImmTACs by Harper et al., extensive human in vitro cellular systems can be used for nonclinical safety assessments [67]. Alternatively, surrogate molecule strategies can be considered. In vivo pharmacology data can be obtained by constructing surrogate molecules that recognize homologous murine target antigens (e.g., murine PRAME peptide). However, functional validation should be performed. The differences in structure and binding characteristics between the surrogate molecule and the clinical candidate molecule, and the limitations of extrapolating results to humans, should be explained.

### 3.3. Pharmacodynamic Studies

ICH S6(R1) recommends pharmacodynamic (PD) assessment for biotechnology-derived products [63]. For soluble TCR-based protein biologics, we propose that in vitro PD studies evaluate target-cell killing activity (EC_50_, maximal efficacy, E:T ratio dependence), T-cell activation markers(CD25, CD69, etc.), and cytokine secretion profiles(IFN-γ, IL-2, IL-6, and TNF-α), based on published experience with tebentafusp and ImmTACs [67]. For example, IFN-γ secretion was detected in a preclinical cellular assay for Tebentafusp. We further propose that in vitro PD experiments include strict HLA-positive/negative and antigen-positive/negative control groups to verify HLA restriction and antigen dependence, respectively [67]. Cell lines with different HLA allele combinations should be included to demonstrate that activity occurs only on HLA/antigen double-positive cells.

In vivo PD evaluations typically employ immunodeficient mouse models bearing human HLA-positive tumor cells combined with the adoptive transfer of human peripheral blood mononuclear cells. Alternatively, ex vivo co-culture systems using patient-derived primary tumor cells and autologous T cells can be used. The limitations of each model in simulating the solid tumor microenvironment (especially the inhibitory effect of tumor-associated macrophages on ImmTAC activity [69]) should be fully discussed.

### 3.4. Toxicology Studies

Toxicology studies for soluble TCR-based protein biologics include acute toxicity studies to assess the maximum tolerated dose or the highest non-severely toxic dose. Cytokine-related acute toxicity (fever, hypotension) and skin reactions (lineage toxicity) should be closely monitored, and repeat-dose toxicity (GLP) studies should be conducted. Key study parameters include cytokine profiles, hematology, liver and kidney function, and systemic pathology. Skin (e.g., related to pigment cells), ophthalmic, and cardiac pathology should also be determined. Genotoxicity, carcinogenicity, and reproductive toxicity studies should be conducted according to the ICH S9 exemption rules and M3(R2) staging principles [64].

Evaluation of pHLA specificity and cross-reactivity is an important component of soluble TCR-based protein biologic research. However, current regulatory guidance, including ICH S6(R1), does not specifically define pHLA cross-reactivity testing for T-cell receptor protein drugs. For affinity-enhanced TCRs, because engineering modifications may alter their peptide recognition repertoire and HLA restriction, target pHLA specificity and non-target pHLA cross-reactivity should be considered core evaluation components. We propose a tiered pHLA cross-reactivity evaluation framework. First, the specific recognition of target pHLA and functional activation by soluble TCR-based protein biologics should be verified. Subsequently, a tiered pHLA cross-reactivity evaluation system should be established. Tier 1 involves in silico computational screening, integrating HLA binding prediction, proteasomal processing prediction, and expression profiling to preliminarily screen candidate cross-reactive peptides, not limited to peptides with high sequence homology to the target peptide [67]. Tier 2 involves alanine scanning and X-scanning to identify key residues involved in TCR binding and to preliminarily map the TCR binding motif, systematically evaluating the degeneracy of peptide recognition and cross-reactivity potential [67,70]. Tier 3 involves functional validation of candidate cross-reactive peptides, using T cell activation assays and target cell cytotoxicity assays to assess whether the candidate cross-reactive peptides exhibit substantial biological activity. Tier 4 involves evaluation of non-target HLA allele recognition, using cell lines that express different HLA alleles and present the target peptide or candidate cross-reactive peptides to assess the risk of cross-recognition [67]. Finally, alloreactivity should be evaluated, with the risk assessed using a mixed lymphocyte reaction (MLR) with allogeneic PBMCs.

Cytokine release syndrome (CRS) is a major clinical safety risk of soluble TCR-based protein biologics (in the phase III study of Tebentafusp, the incidence of CRS was 89%, mostly grade 1–2 [61]). For CRS risk assessment, in vitro cytokine release assays (CRA) should be performed using PBMCs co-cultured with HLA/antigen double-positive target cells to quantitatively evaluate concentration-dependent cytokine release curves and determine the threshold concentration for CRS risk.

### 3.5. Pharmacokinetics and Immunogenicity

Pharmacokinetic (PK) assessment is needed for soluble TCR-based protein biologics. However, ICH S6(R1) notes that it is difficult to establish uniform PK testing guidelines for biotechnology-derived products [63]; therefore, PK evaluation should be designed on a case-by-case, science-based basis. For soluble TCR-based protein biologics, we propose that PK studies consider the molecular format (Fc-free vs. half-life–extended formats), target-mediated drug disposition, and the impact of anti-drug antibodies, rather than following a fixed PK template. Fc-free soluble TCR fusion proteins have extremely short half-lives (requiring daily to weekly dosing), whereas dosing intervals are longer for Fc-fused or half-life extended formats (e.g., TCER^®^). Receptor occupancy-pharmacokinetic/pharmacodynamic relationship models should be established to optimize dose regimens.

ICH S6(R1) states that immunogenicity assessment aids in the interpretation of study results and the design of subsequent studies [63]. Engineered soluble TCR sequences may contain non-human sequences, which have inherent immunogenicity risks. We propose that early immunogenicity risk assessment be performed using in silico T-cell epitope prediction (e.g., EpiMatrix tool) and in vitro human peripheral blood mononuclear cell (PBMC) proliferation assays to guide the optimization of sequence humanization. We further propose that the impact of anti-drug antibodies on pharmacokinetics (particularly more significant for Fc-free formats with short half-lives) should be systematically monitored in toxicology studies.

## 4. Clinical Research on Soluble TCR-Based Protein Biologics

### 4.1. Tebentafusp

Tebentafusp (tebentafusp-tebn; Kimmtrak^®^), which is already on the market, is a first-generation soluble TCR-based protein biologic developed by Immunocore to treat uveal melanoma. Tebentafusp is an ImmTAC, consisting of a high-affinity TCR targeting the gp100 peptide presented by HLA-A*02:01 fused with an anti-CD3 scFv [61]. In a Phase I/II clinical trial of tebentafusp (NCT02570308), serum levels of inflammatory cytokines (IFN-γ, TNF-α, IL-6, etc.), chemokines, and intratumoral CD3+ and CD8+ T-cell counts increased in patients after treatment, demonstrating the redirection of T cells and activation of the immune system in response to tebentafusp. In patients with uveal melanoma, multiple administrations of tebentafusp at the recommended dose did not induce tebentafusp accumulation, and the median terminal half-life of tebentafusp was 7.5 h [71]. In a Phase III clinical trial (NCT03070392), HLA-A*02:01-positive patients with untreated metastatic uveal melanoma received intravenous tebentafusp in the experimental group, and the control group received alternative therapies (pembrolizumab, ipilimumab, or dacarbazine). Tebentafusp significantly improved outcomes (hazard ratio [HR] = 0.51 at the first interim analysis). The 1-year overall survival (OS) rate, disease control rate (DCR), progression-free survival (PFS) rate, and objective response rate (ORR) were all higher in the tebentafusp group than in the control group [61]. In a subsequent clinical trial (NCT02570308), patients treated with tebentafusp exhibited durable tumor shrinkage and reduced tumor growth rates. The 1-year, 2-year, and 3-year OS rates for tebentafusp were 72%, 45%, and 27%, respectively [72]. The success of tebentafusp validates the feasibility and potential of soluble TCR-based protein biologics for cancer treatment.

### 4.2. Brenetafusp

Brenetafusp (IMC-F106C), the first ImmTAC targeting PRAME, was developed by Immunocore to treat melanoma. Brenetafusp is currently in Phase III clinical development. Brenetafusp consists of a high-affinity TCR targeting the PRAME peptide presented by HLA-A*02:01 and an anti-CD3 scFv portion. A Phase I clinical trial (NCT04262466) reported promising disease control by brenetafusp monotherapy and combination therapy with anti-PD1 in cutaneous melanoma [73]. Subsequent Phase III clinical trials (NCT06112314) will continue to evaluate the potential of brenetafusp in combination with the anti-PD1 ICI nivolumab to improve PFS, OS, and ORR [74]. The latest clinical trial (NCT07686367) will test the therapeutic effect of Brenetafusp in rare cancers [75].

### 4.3. IMA402

IMA402 is a PRAME- and CD3-targeting bispecific soluble TCR-based protein biologic developed by Immatics. IMA402 is currently in Phase I/II clinical development. IMA402 is a TCER that simultaneously targets the PRAME peptide presented by HLA-A*02:01 and CD3. IMA402 elicits antitumor activity in solid tumors, including cutaneous melanoma (uveal melanoma) and ovarian cancer.

A Phase I/II clinical trial (NCT05958121) is currently underway to evaluate the safety and tolerability of IMA402, as well as its antitumor activity as monotherapy and in combination therapy [76]. IMA402 has shown promising clinical potential.

### 4.4. IMA401

IMA401 is a soluble TCR-based protein biologic with a TCER structure developed by Immatics to treat solid tumors, squamous non-small cell lung cancer, gastric cancer, and melanoma. IMA401 simultaneously targets the MAGE-A4/8 peptide presented by HLA-A*02:01 and CD3. The HLA-A*02:01-presented MAGE-A4/8 target peptide shows presentation levels in tumor cells at least 5-fold higher than those of a commonly used epitope, while being virtually absent from normal tissues. IFNγ stimulation can increase its presentation by approximately 70-fold. The MAGE-A4/8 target gene is widely expressed in solid tumors, especially in squamous non-small cell lung cancer (sqNSCLC) and head and neck squamous cell carcinoma (HNSCC) [29].

A Phase I clinical trial (NCT05359445) evaluated IMA401 in patients with relapsed/refractory solid tumors [77]. In IMA401 monotherapy and pembrolizumab combination therapy, the most common adverse event was low-grade CRS (mostly grade 1–2, with no grade ≥ 3 events). IMA401 demonstrated promising antitumor activity both as monotherapy and in combination with pembrolizumab. In patients with several different tumor indications, the duration of response (DOR) exceeded 6 months in most cases, and the longest response lasted more than 2.5 years, demonstrating significant objective responses [29].

### 4.5. IMC-M113V

IMC-M113V, which was developed by Immunocore, is the first TCR bispecific immunotherapy targeting HIV. IMC-M113V simultaneously targets the HIV Gag peptide (SLYNTVATL, residues 77–85) presented by HLA-A*02:01 and CD3. IMC-M113V is in Phase I/II clinical development. The ImmTAV drug against the Gag SL9 epitope is well tolerated and exhibits dose-dependent viral control after interrupting antiretroviral therapy [78]. In the Phase I/II STRIVE trial, IMC-M113V was well tolerated, with no serious adverse events or CRS of grade 2 or higher. The most common adverse event was grade 1 pyrexia, associated with T-cell activation. Treatment with IMC-M113V reduced viral levels in the blood of AIDS patients. The PD activity of IMC-M113V is dose-dependent and strongest in participants with highly IMC-M113V-sensitive Gag77–85 variant sequences. Twenty percent of patients achieved viral control after antiretroviral therapy interruption, and signs of viral reservoir reduction were observed [78]. Thus, soluble TCR-based protein biologics are a promising new strategy for targeting the functional cure of HIV.

### 4.6. CLSP-1025

CLSP-1025 was developed by Clasp Therapeutics to treat colorectal cancer, solid tumors, lung cancer, and ovarian epithelial cancer. CLSP-1025 is a pHLAre molecule targeting p53-R175H and CD3 and is in Phase I clinical development (NCT06778863). CLSP-1025 is a half-life extended single-chain diabody-IgG4 Fc fusion protein that binds to the 168–176 peptide of p53-R175H presented by HLA-A*02:01 on cancer cells [79]. The p53-R175H mutation occurs early in tumor development and is present in multiple solid tumor cells. Thus, the p53-R175H mutation is an ideal target for immune system attack [80]. Clinical research on CLSP-1025 is ongoing.

### 4.7. CRPA1A2

CRPA1A2, which received IND permission from the FDA in December 2025, is a soluble TCR-based protein biologic developed by CorreGene to treat solid tumors. CRPA1A2 targets MAGEA1, a cancer/testis antigen, and CD3 [81]. MAGE-A1 is highly expressed in solid tumors such as liver cancer, lung squamous cell carcinoma, esophageal cancer, head and neck cancer, and gastric cancer, but is not expressed in normal human somatic cells [82]. CRPA1A2 is currently entering Phase I clinical studies (NCT07583316).

### 4.8. Other Soluble TCR-Based Protein Biologics

Several ImmTAX-class drugs developed by Immunocore are in the early clinical research stages. For example, IMC-R117C, the first immunotherapeutic agent simultaneously targeting PIWIL1 and CD3 for colorectal cancer, esophageal cancer, and gastric cancer, is currently in Phase I/II clinical development (NCT06840119) [83]. IMC-P115C, targeting PRAME and CD3 for advanced PRAME-positive solid tumors, is currently in Phase I/II clinical development (NCT07156136) [84]. IMC-C103C, which simultaneously targets MAGEA4 and CD3, is being developed to treat solid tumors and ovarian epithelial cancer and is currently in Phase I/II clinical development (NCT03973333) [85]. IMC-I109V, simultaneously targeting Env and CD3 to treat hepatitis B virus infection, is currently in Phase I clinical development (NCT05867056) [86]. Thus, the clinical development of soluble TCR-based protein biologics is steadily advancing.

## 5. Control Strategy for Soluble TCR-Based Protein Biologics

Soluble TCR-based protein biologics share attributes with antibody drugs. Quality specifications based on the molecular structure and mechanism of action of soluble TCR-based protein biologics should include the entire soluble TCR-based protein biologic and its key components, the anti-pHLA portion and the anti-CD3 portion. Similar to bispecific antibodies such as TCEs, the structure of soluble TCR-based protein biologics can vary considerably. Their critical quality attributes should be determined based on those of similar bispecific antibodies. Appropriate techniques and analytical methods based on structure, physicochemical properties, and biological activity should be developed, while considering design principles and the manufacturing process [87].

### 5.1. Manufacturing Process and Process Control

Adequate process design and an understanding of process capability are important components of developing a controllable and reproducible manufacturing process to ensure the production of a drug product that meets quality specifications [87]. The manufacturing process should consider the structural characteristics of the drug. Soluble TCR-based protein biologics contain scFv structures, and some include Fc fragments. The molecular structures of TCR drugs are complex and diverse. For example, Fc-free soluble TCR-based protein biologics, such as ImmTAC structures, and soluble TCR-based protein biologics containing Fc fragments, such as TCER and CorEngager, require attention to the manufacturing processes of the different structural types. Process control includes product quality attributes and process parameters. The control and removal validation of process-specific product-related impurities are important for the manufacturing of soluble TCR-based protein biologics. These control measures should ensure that the product meets established quality attributes, such as product-related degradation fragments and other impurities. During the entire soluble TCR-based protein biologic manufacturing process, particular attention should be given to homodimers, mismatched disulfide bonds, half-antibodies, and free TCR structures resulting from the asymmetric architecture. Process validation should demonstrate the capacity to remove impurities unique to the soluble TCR-based protein biologic product. Throughout the manufacturing process, comprehensive process validation is required through multiple consecutive batches (at least three batches) to assess process reproducibility and consistency. For process validation batches, intermediates and drug products should be extensively tested, analyzed, and characterized more than routine batches to establish routine batch process controls and release specifications [87,88].

### 5.2. Characterization of Soluble TCR-Based Protein Biologics

#### 5.2.1. Primary Structure

The soluble TCR-based protein biologic structure should be sequenced, usually with LC-MS. The drug is specifically digested with proteases into characteristic peptides. The individual peptide fragments are separated by liquid chromatography and injected into a second mass spectrometer to obtain the primary structure of the drug. Disulfide bonds in soluble TCR-based protein biologics stabilize the structure and maintain activity [25]. The number, position, and integrity of disulfide bonds should be determined, and disulfide bond mismatches should be identified. Class, subclass, and chain composition analysis should be performed, and the presence or absence of glycosylation sites in the Fc region should be determined.

#### 5.2.2. Identification

Identification of soluble TCR-based protein biologics includes peptide mapping and charge heterogeneity analyses. When establishing quality control requirements for soluble TCR-based protein biologics, the protein amino acid sequence should be confirmed, and relevant modifications should be identified and quantified. Routine peptide mapping is an important quality control method to characterize soluble TCR-based protein biologics. Most modifications can alter the surface charge of the antibody structure by changing the number or structure of charged groups, resulting in charge heterogeneity. Therefore, reasonable methods for analyzing the charge heterogeneity of soluble TCR-based protein biologics should be established. The soluble TCR-based protein biologic charge variants can be separated, based on the isoelectric points, and identified by capillary electrophoresis methods, including imaged capillary isoelectric focusing (icIEF) or capillary isoelectric focusing [87].

#### 5.2.3. Biological Activity

Biological activity is a critical quality attribute of soluble TCR-based protein biologics. Biological activity assay methods should be established to evaluate the efficacy, stability, and batch-to-batch consistency of soluble TCR-based protein biologics. The biological effects of soluble TCR-based protein biologics involve multiple steps, including bispecific binding to targets, T-cell activation, and target cell killing, making biological activity determinations challenging [87]. Thus, multiple independent potency assays are needed to confirm the functionality of each target, and the specificity of method validation should also be addressed during this process.

After pHLA and TCR interact, various proteins are activated and participate in signal transduction, activating nuclear factors of activated T cells (NFAT) to induce luciferase gene expression in a dose-dependent manner. Therefore, reporter gene T-cell activation assays are often used for determining and evaluating biological activity, instead of single-arm binding SPR assays, cell proliferation assays, and bridging enzyme-linked immunosorbent assay (ELISA) methods [89]. Reporter gene activity assays employ engineered T cells, which express luciferase after T-cell activation, utilizing Jurkat-NFAT reporter cells expressing CD3 and T2 cells loaded with the target peptide. Cytokine (e.g., IFN-γ, granzyme B, or IL-2) detection is performed after killing activity assays. Flow cytometry is used to determine if soluble TCR-based protein biologics dose-dependently increase the expression of the T-cell activation markers, CD25 and CD69 [90], followed by ELISA or bridging ELISA.

**Figure 5:**
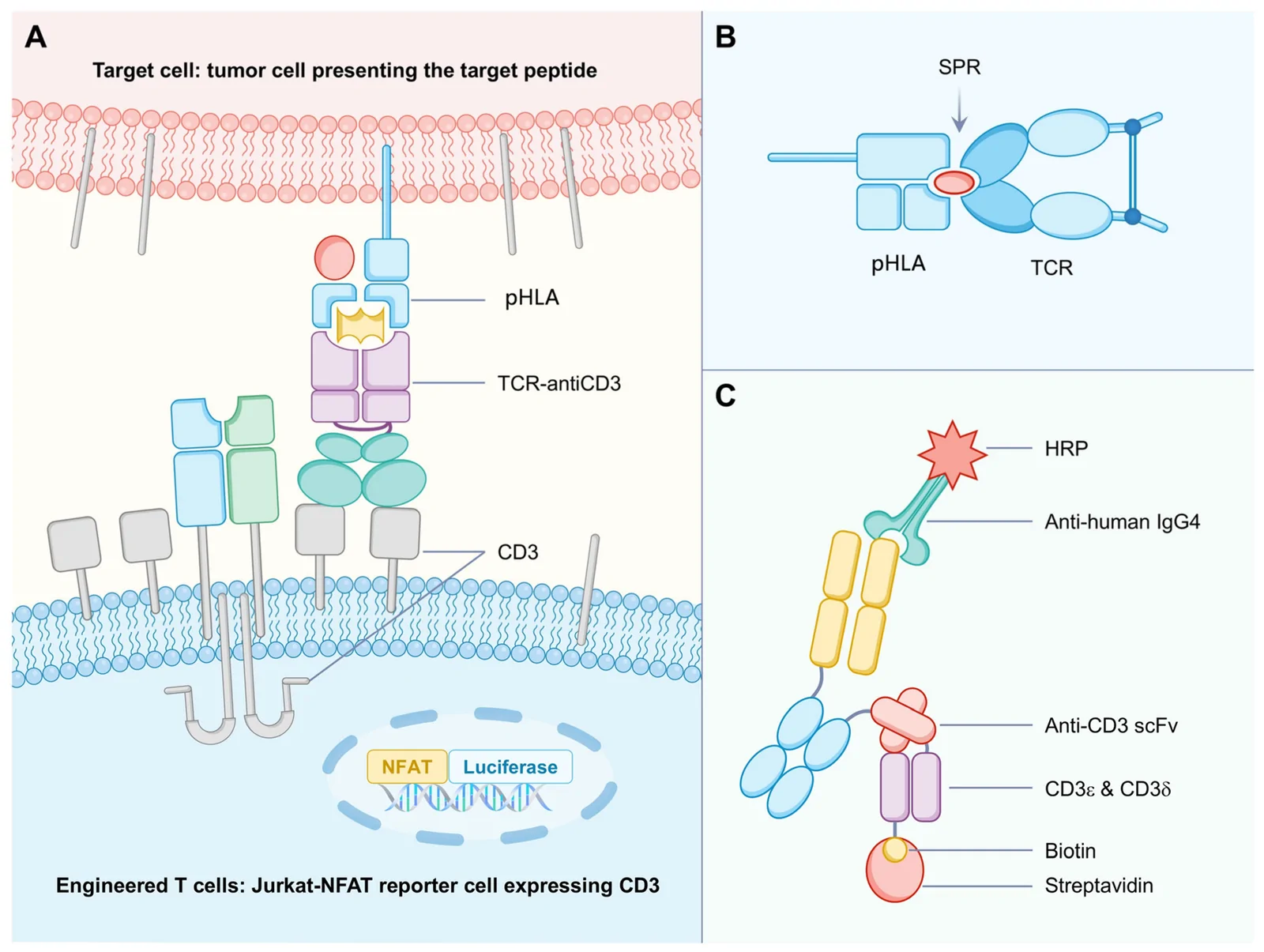
Mechanism and method of bioassay. Biological activity was measured using a reporter gene assay (**A**). Surface Plasmon Resonance (SPR) was used to characterize the binding of the TCR to pHLA and of the anti-CD3 domain to CD3 (**C**), and the binding activity was measured by enzyme-linked immunosorbent assay (ELISA) (**B**). NFAT, nuclear factor of activated T cells; HRP, horseradish peroxidase.

After determining the overall activity of the soluble TCR-based protein biologic, the recognition processes at the pHLA and CD3 ends should be determined separately. During the pHLA recognition process at the TCR end, tumor cells presenting the target peptide are used as target cells to interact with the TCR end for HLA-peptide complex capture analysis, and Surface Plasmon Resonance (SPR) is used to determine the binding activity of the TCR end to pHLA [91]. For the recognition process at the CD3 end, CD3δε-HRP detection or Anti-Vl detection is often used [90]. The mechanism and method of bioassay are shown in Figure 5.

#### 5.2.4. Purity

Purity is an important aspect of quality control for soluble TCR-based protein biologics. Purity reflects the stability of the manufacturing process and product consistency and is a core consideration for regulatory agency approval. Purity is related to the safety and efficacy of soluble TCR-based protein biologics. Even trace impurities can trigger immunogenic reactions or other adverse effects. Therefore, establishing a reasonable quality specification for purity is important [87,88]. The biological function of soluble TCR-based protein biologics depends on the integrity of their structure, and degradation forms (e.g., fragments, aggregates) directly affect the ability of the soluble TCR-based protein biologic to bind antigens and mediate effects [87]. Size exclusion chromatography, ion exchange chromatography, size exclusion high-performance liquid chromatography, icIEF, hydrophobic interaction chromatography, reversed-phase high-performance liquid chromatography, and capillary electrophoresis can be used for determining purity.

#### 5.2.5. Homodimers

Soluble TCR-based protein biologics have complex structures and are difficult to purify. Soluble TCR-based protein biologics are more prone to higher-order structure mispairing than conventional monoclonal antibodies. Therefore, impurity detection should include monitoring mispaired products, homodimers, fragments, and aggregates. This is particularly important for effector cell-engaging constructs, in which homodimers formed by the anti-CD3 or anti-Fc engaging arm may induce cytokine release [62]. Soluble TCR-based protein biologics have an asymmetric structure. Homodimer impurities generated during production may pose challenges to product manufacturing processes and quality control. Therefore, establishing reasonable analytical methods for detecting homodimers is very important in the quality control of soluble TCR-based protein biologics. Homodimers should be evaluated as critical quality attributes. Capillary electrophoresis and liquid chromatography can be used to determine homodimer content, and reasonable specification limits should be established [87].

#### 5.2.6. Residuals

Process-related impurities in soluble TCR-based protein biologics include host cell proteins (HCPs), host cell DNA (HCD), and Protein A. Reasonable methods should be established to analyze residual substances during the production of soluble TCR-based protein biologics. ELISAs can be used to determine residual amounts of HCPs and Protein A, and quantitative PCR can be used to determine residual amounts of HCD. Other process impurities may enter the final product from various sources. Each impurity should be evaluated separately and controlled at safe levels based on clinical experience and regulatory requirements [87].

### 5.3. Stability Studies

Stability studies are important throughout the drug development process and are the basis for determining product shelf life. Stability studies can be used to justify the manufacturing process, formulation, and packaging material decisions and are also the foundation for product quality specifications. The basic principles of stability studies for soluble TCR-based protein biologics are similar to the requirements for antibody therapeutic products. Study protocols should be designed based on the characteristics of the soluble TCR-based protein biologic, clinical application requirements, and specific storage, packaging, and transportation conditions to ensure the stability and safety of the drug throughout the supply chain. Physical, chemical, and microbial stability studies should also be conducted on intermediates that are temporarily stored during the manufacturing process to verify their appropriate storage conditions and duration [88]. These studies reveal the changes in soluble TCR-based protein biologics under different environments and provide critical data for optimizing manufacturing and storage processes.

### 5.4. Quality Specifications

The structure of soluble TCR-based protein biologics is similar to the structure of bispecific antibodies. Therefore, the quality control methods for bispecific antibodies can be referenced to identify the physicochemical properties and analyze and detect key drug components of soluble TCR-based protein biologics. Quality control requirements can be expanded based on the unique attributes of soluble TCR-based protein biologics, with particular attention to potency (e.g., pHLA binding, CD3 binding, bifunctional bridging, T cell activation) and product-related impurities (e.g., aggregates, fragments, mispaired species). Quality specifications should be established based on drug characterization and quality studies. The core principle of quality specifications is ensuring, through adequate testing, that the product meets established requirements for identity, purity, safety, and efficacy. Analytical methods should be fully developed and validated, especially for newly developed methods. Tests to assess the quality of soluble TCR-based protein biologics should be based on product quality studies, a thorough understanding of the manufacturing process and clinical indications, and consideration of the product characteristics and current scientific understanding and consensus [92]. As research progresses from preclinical to clinical stages, process-related information should accumulate, and analytical methods should be progressively refined to support quality control requirements at each stage.

Quality specifications for soluble TCR-based protein biologics should include attributes such as color, clarity, osmolality, pH, fill volume, visible particulates, and subvisible particles [87]. Safety specifications should include the presence of viruses, endotoxins, and abnormal toxicity [93]. Specific test items should be determined based on the final product type, manufacturing process, stability studies, and risk assessment. Reference standards, which should be representative and traceable, are very important for ensuring the consistency and accuracy of test results. Risks originating from starting cells, intermediates, and materials or equipment in contact with the product should be assessed. The homogeneity, stability, and safety of the drug substance during the manufacturing process should also be considered. Therefore, the compatibility of components in contact with the product and the integrity of the closed system used for the drug substance should be monitored. These risks should be carefully considered when establishing quality control for soluble TCR-based protein biologics, and proactive monitoring strategies should be implemented.

## 6. Shortcomings and Challenges

**Figure 6:**
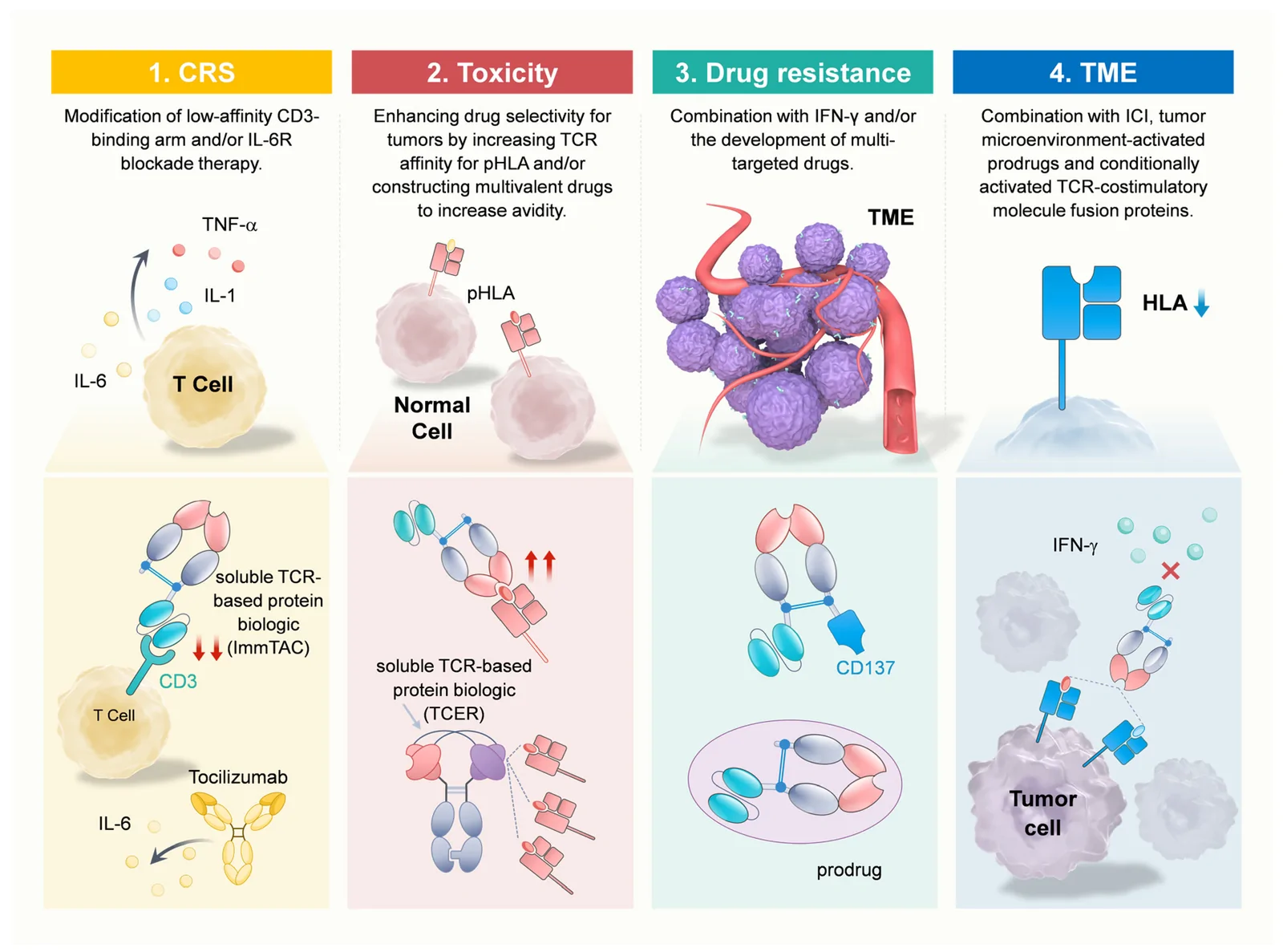
Soluble TCR-based protein biologic challenges and areas for improvement. For different challenges, strategies can be drawn from clinical experience with soluble TCR-based protein biologic or from experience with other therapeutic modalities. IL, Interleukin; TME, tumor microenvironment; IFN, interferon.

Soluble TCR-based protein biologics can simultaneously target tumor targets and TCRs, redirecting immune T cells to tumor cells and utilizing the human body’s own immunity to achieve tumor killing via T-cell activation. Soluble TCR-based protein biologics can utilize HLA to present intracellular tumor antigens on the cell surface, expanding the range of selectable targets and improving drug targeting. However, soluble TCR-based protein biologics also have shortcomings. The soluble TCR-based protein biologic challenges and shortcomings are shown in Figure 6.

### 6.1. CRS

CRS is an uncontrolled systemic inflammatory reaction triggered by massive T-cell activation. T cells are activated during treatment with soluble TCR-based protein biologics. Activated T cells release large amounts of inflammatory cytokines (e.g., TNF-α, IFN-γ, IL-1, and IL-6), which can create a cytokine storm. The cytokine storm causes symptoms ranging from mild flu-like symptoms (fever, fatigue, myalgia) to severe, potentially fatal multi-organ failure [94]. IL-6 triggers a series of systemic symptoms, including fever, vascular leakage, and acute phase reactions. The actions of IL-1 may be upstream of IL-6 [95]. Higher binding affinity to CD3 leads to stronger T-cell activation and better tumor killing in preclinical models. However, high-affinity binding also leads to high levels of cytokine release, resulting in a high incidence and severity of clinical CRS and a narrow therapeutic window [96].

### 6.2. Toxicity

More than 10 antigens can be presented by HLA [15,97], and the estimated number of available TCRs is 10 [11,98]. To prevent the immune system from becoming too large to function, TCRs cannot be absolutely specific, making cross-reactivity inevitable. Therefore, soluble TCR-based protein biologics may cause toxicity by interacting with pHLA at non-target sites due to cross-reactivity during target recognition [99,100]. Additionally, many tumor targets are also expressed at low levels in normal tissues, leading to on-target/off-tumor toxicity through the interaction of soluble TCR-based protein biologics with target antigens in normal tissues. For example, soluble TCR-based protein biologics targeting Gp100 (e.g., tebentafusp) often cause on-target/off-tumor toxicity, including rash, pruritus, and hair depigmentation, due to target expression on normal skin melanocytes [61]. According to the results of the IMCgp100 Phase I/II study, the incidence of rash during tebentafusp treatment is approximately 83% [61]. The skin toxicity caused by IMCgp100 is due to T-cell infiltration and killing of melanocytes, consistent with the antitumor mechanism. Notably, the occurrence of skin toxicity is associated with better treatment response and survival benefits. TCRm may have off-target toxicity due to sequence homology, which is distinct from conventional antibodies. To recognize pHLA, the Fab segment of TCRm uses most of its binding free energy to bind to the invariant HLA framework; typically, only 3–5 key amino acids are involved in recognizing peptide-specific residues, posing a risk of off-target toxicity due to recognition of peptides with similar sequences [30].

### 6.3. Drug Resistance

The efficacy of tumor immunotherapy depends on the immune system recognizing tumor cells. Therefore, antigen loss or downregulation, T-cell exhaustion, and abnormal intrinsic signaling pathways in tumor cells can cause drug resistance. The efficacy of soluble TCR-based protein biologics with soluble TCR structures depends entirely on the expression of HLA class I molecules on the target cell surface. Tumor cells often evade immune recognition by downregulating HLA class I expression via genetic mutations, epigenetic silencing, or signaling pathway defects [101,102]. Therefore, if the target on the tumor cell cannot be presented due to HLA deficiency, the T cells cannot recognize the target, and treatment is ineffective even if T cells are activated. Resistance can also develop due to tumor heterogeneity. Tumor antigen heterogeneity includes spatial heterogeneity and temporal heterogeneity. Spatial heterogeneity refers to the uneven distribution of tumor cell subpopulations in different regions of the primary tumor or among different metastatic sites at the same time. Temporal heterogeneity refers to dynamic changes in the molecular characteristics of the tumor over time, especially under treatment pressure [103]. Incorporating heterogeneity as a routine variable into study design can reduce drug resistance.

### 6.4. Impact of the Tumor Microenvironment

The tumor microenvironment (TME) is a highly heterogeneous and dynamic system composed of various cellular, extracellular, and soluble components that influence tumor progression and immune regulation. The cellular components include malignant cells, cancer-associated fibroblasts, and immune cells such as tumor-associated macrophages, natural killer cells, myeloid progenitor cells, myeloid-derived suppressor cells (MDSCs), effector and regulatory T cells (Tregs), dendritic cells, and neutrophils. Cancer cells and immune cells within the TME produce secreted factors, including cytokines, chemokines, and various signaling molecules. The efficacy of soluble TCR-based protein biologics in tumors is influenced by the TME environment, including functionally exhausted T cells, immunosuppressive factors such as IL-10 and TGF-β, insufficient T-cell infiltration, and Tregs. Immunosuppressive cells and physical barriers in the TME also limit T-cell infiltration. Even if soluble TCR-based protein biologics successfully bind to targets, the T cell-mediated cytotoxicity/redirected killing effects will be significantly reduced if the TME is filled with immunosuppressive cells such as Tregs and MDSCs [104]. The immunological characteristics of the TME should also be considered in the context of organ-specific factors, which may vary depending on the organ. The TME has clinical implications for soluble TCR-based protein biologics; different disease sites within the same patient may require different treatment strategies tailored to their unique microenvironments.

## 7. Conclusions and Outlook

The research and development of soluble TCR-based protein biologics that precisely target pHLA complexes to recruit T cells for tumor killing is highly challenging. Among the strategies addressing the challenges in soluble TCR-based protein biologic development, some have entered clinical development or been validated preclinically in soluble TCR-based protein biologics, whereas others are derived from the experience of CAR-T, TCEs, and other immunotherapies and still require further validation in soluble TCR-based protein biologics.

To address the risk of CRS, optimization strategies from TCEs can be adopted, including engineering low-affinity CD3-binding arms to attenuate excessive T-cell activation while preserving antitumor efficacy [105]. IL-6R blockade (e.g., tocilizumab) is the first-line treatment for managing CRS [106]; experience blocking CRS can be derived from the CAR-T and TCE fields. Notably, the potential advantage of IL-1R blockade (e.g., Anakinra) for immune effector cell-associated neurotoxicity syndrome (ICANS) has been demonstrated in CAR-T therapy [107]. The incidence of ICANS induced by soluble TCR-based protein biologics is relatively low; therefore, the advantages of IL-1R blockade for soluble TCR-based protein biologics require further study.

Several preclinically validated strategies can be employed to optimize the therapeutic window and improve the tumor selectivity of soluble TCR-based protein biologics. First, TCR affinity for pHLA can be increased through techniques such as phage display, utilizing the affinity threshold effect to preferentially kill high-expressing tumor cells. Second, multivalent formats (e.g., 2 + 1 TCER) can be developed to increase avidity and enhance binding stability [29]. Accurately and rapidly screening patients most likely to benefit from a specific soluble TCR-based protein biologic therapy may reduce toxicity and improve drug selectivity. For example, for soluble TCR-based protein biologics targeting pHLA, patient HLA typing and specific antigen expression are necessary.

To avoid resistance to soluble TCR-based protein biologics, combining the soluble TCR-based protein biologic with IFN-γ can upregulate HLA Class I expression on tumor cells [108], increasing pHLA target density and potentially enhancing the efficacy of soluble TCR-based protein biologics. However, IFN-γ may induce the immunosuppressive side effect of PD-L1 expression [109], and this strategy requires further clinical validation. Additionally, chemotherapy and targeted drugs may enhance the efficacy of soluble TCR-based protein biologics by inducing immunogenic cell death or upregulating HLA Class I expression, but specific combination regimens should consider the risk of soluble TCR-based protein biologics-induced CRS, and systematic clinical validation is currently lacking.

Combining soluble TCR-based protein biologics with immune checkpoint inhibitors may be a feasible option for immunosuppression in the tumor microenvironment. Soluble TCR-based protein biologics can reverse Treg-mediated immunosuppression [65], while ICIs relieve PD-1/PD-L1 pathway inhibition [110], synergistically enhancing antitumor immunity. The available clinical data up to 2025 show that the combination of IMA401 (MAGEA4/8 TCER) with pembrolizumab is feasible in patients who have progressed on prior ICI therapy [29]. Developing drug molecules capable of counteracting or remodeling the TME based on experience with other therapeutic modalities is also an important direction. For example, informed by experience with XTX101, an anti-human CTLA-4 monoclonal antibody (mAb) covalently linked to masking peptides, and with the IL-2 prodrug WTX-124 [111,112], tumor microenvironment-activated prodrugs could be designed to restrict drug activation to the local site via tumor-specific protease cleavage or antigen binding, thereby reducing systemic toxicity. Another innovative strategy is designing conditionally activating TCR-co-stimulatory molecule fusion proteins; using TCR recognition of pHLA complexes to anchor in the tumor and locally provide CD137 (4-1BB) co-stimulatory signals (signal 2), preferentially activating antigen-specific T cells at the tumor site rather than systemically activating all T cells, balancing efficacy and safety. Unlike CD3-targeting TCEs (providing signal 1), such molecules require endogenous TCR signaling or combination with other therapies that provide signal 1 (e.g., TCEs or vaccines). Currently, TCR-CD137 fusion proteins have not yet entered the clinic but have been validated in TCE + CD137 bispecific antibody combination therapies (e.g., FAP-4-1BBL combined with CEA-TCB) [113], demonstrating the potential to enhance memory T-cell formation and reduce exhaustion. These approaches currently require further validation in the design of TCR-based protein therapeutics.

As a novel class of precision cancer therapeutics, soluble TCR-based protein biologics are at the forefront of global research and development. Innovative soluble TCR-based protein biologics for multiple indications are showing encouraging results in clinical studies. By recognizing pHLA complexes, soluble TCR-based protein biologics can target intracellular antigenic peptides presented by HLA, expanding the targetable tumor antigen spectrum. The top priorities in clinical research include how to refine toxicity management to reduce on-target toxicities such as CRS while optimizing efficacy; how to expand targets to discover more public neoantigens such as *KRAS* G12V and MAGE-A4; and how to optimally combine soluble TCR-based protein biologic with existing therapies (e.g., checkpoint inhibitors) to achieve synergistic effects and overcome resistance. An increasing number of soluble TCR-based protein biologics have entered clinical research stages. Tebentafusp significantly prolongs overall survival in patients with uveal melanoma, and IMA401, IMA402, and other next-generation molecules exhibit preliminary efficacy in solid tumors. Thus, soluble TCR-based protein biologics are gradually validating their unique advantage in targeting intracellular antigens, opening new directions for cancer immunotherapy.

## Figures and Tables

**Table 1:** Soluble TCR-based protein biologics in the global clinical trial pipelines.

Drugs	Company	Disease	Antigen	Phase	Type
Tebentafusp	Immunocore	Uveal Melanoma	CD3 × gp100	Approved	ImmTAC
Brenetafusp	Immunocore	Melanoma	CD3 × PRAME	III	ImmTAC
IMC-R117C	Immunocore	Colorectal cancer; GI cancer	CD3 × PIWIL1	I/II	ImmTAC
IMC-P115C	Immunocore	Solid tumor	CD3 × PRAME	I/II	ImmTAC
IMA402	Immatics	Solid tumor; Cutaneous melanoma; Uveal melanoma	CD3 × PRAME	I/II	TCER
IMC-M113V	Immunocore	HIV	CD3 × Gag	I/II	ImmTAV
IMC-C103C	Immunocore	Solid tumor; Epithelial ovarian cancer	CD3 × MAGEA4	I/II	ImmTAC
IMCnyeso	Immunocore	Solid tumor	CD3 × NY-ESO-1	I/II	ImmTAC
CLSP-1025	Clasp Therapeutics	Colorectal cancer; Solid tumor; Lung cancer	CD3 × p53-R175H	I	pHLAre
IMA401	Immatics	Solid tumor; Squamous non-small cell lung cancer; GI cancer	CD3 × MAGEA4/A8	I	TCER
IMC-I109V	Immunocore	HBV	CD3 × Env	I	ImmTAV
CRPA1A2	Corregene	Solid tumor	CD3 × MAGEA1	IND approval	CorEngager

Note: ImmTAC, Immune mobilizing monoclonal T-cell receptors Against Cancer; ImmTAV, Immune mobilizing monoclonal T cell receptors Against Virus; TCER, T cell Engaging Receptors; pHLAre, precise HLA redirecting engagers.

**Table 2:** Comparison of soluble TCR-based protein biologics and related T-cell redirecting modalities.

Molecule/Construct	Type	Structure	Target Recognition	HLA Dependence	Mechanism of Action	Representative Drugs
Endogenous TCR	Natural receptor, not a therapeutic drug	αβ TCR heterodimer on T cells	pHLA complex	Yes	T-cell activation via CD3 signaling	None (natural receptor)
TCR-TCE	Soluble TCR-based protein biologic	Soluble high-affinity TCR fused to an anti-CD3 binding domain	pHLA complex	Yes	Binds pHLA+ tumor cells and CD3+ T cells; redirects T cells to kill tumor cells	Tebentafusp, brenetafusp
TCRm	Soluble TCR-based protein biologic	Antibody or antibody fragment engineered with TCR-like CDRs	pHLA complex	Yes	Binds pHLA; may mediate antibody effector functions or T-cell redirection if bispecific	Preclinical PRAME TCR-MIMIC (Gilead Sciences)
TCE	Not a soluble TCR-based protein biologic	Bispecific antibody	Surface TAA	No	Binds TAA on tumor cells and CD3; HLA-independent T-cell redirection	Blinatumoma, catumaxomab, tarlatamab

**Table 3:** Assessment and recommendation framework for nonclinical assays.

Assessment	Recommendation Nonclinical Assays	Purpose	Regulatory Basis	Classification
Relevant species assessment	Assess pHLA-recognition and CD3-binding domains separately; alternative strategies if no relevant species	Identify pharmacologically relevant species	ICH S6(R1)	Regulatory requirement + authors’ proposal
PD (in vitro/in vivo)	In vitro: killing, activation markers, cytokines; HLA/antigen controls; in vivo: human PBMC + HLA-positive tumor models	Verify HLA restriction, antigen dependence, potency, and in vivo activity	ICH S6(R1)	Regulatory requirement + Tebentafusp/ImmTAC experience + authors’ proposal
General toxicology studies	Acute toxicity (MTD/HNSTD); repeat-dose GLP; cytokines, hematology, pathology; genotox/carcino/repro	Determine toxicity, target organs, reversibility, and safe dose	ICH S9; ICH S6(R1)	Regulatory requirement + authors’ proposal
pHLA specificity and cross-reactivity	Tiered framework: in silico; alanine/X-scan; functional validation; HLA alleles; MLR.	Assess peptide degeneracy, off-target pHLA, HLA cross-recognition, and alloreactivity	ICH does not specifically define	Authors’ proposal
CRS risk assessment	In vitro CRA: PBMCs + HLA/antigen double-positive target cells; determine CRS threshold.	Determine the threshold concentration for CRS risk to inform dose selection	ICH S6(R1)	Authors’ proposal + Tebentafusp/ImmTAC experience
PK assessment	PK/TK; consider format, TMDD, ADA; receptor occupancy–PK/PD models.	Support dose selection and dosing interval	ICH S6(R1)	Regulatory requirement + authors’ proposal
Immunogenicity assessment	In silico T-cell epitope prediction; PBMC proliferation; monitor ADA impact on PK	Aid interpretation, guide sequence humanization	ICH S6(R1)	Regulatory requirement + authors’ proposal

Note: PD, pharmacodynamics; PBMC, peripheral blood mononuclear cells; MTD, maximum tolerated dose; HNSTD, highest non-severely toxic dose; MLR, mixed lymphocyte reaction; CRS, cytokine release syndrome; CRA, cytokine release assay; PK, pharmacokinetics; TK, toxicokinetics; TMDD, target-mediated drug disposition; ADA, anti-drug antibodies; GLP, good laboratory practice.

## Data Availability

No new data were created or analyzed in this study. Data sharing is not applicable.
